# Genetics and Clinical Features of Noncompaction Cardiomyopathy in the Fetal Population

**DOI:** 10.3389/fcvm.2020.617561

**Published:** 2021-01-20

**Authors:** Hairui Sun, Xiaoyan Hao, Xin Wang, Xiaoxue Zhou, Ye Zhang, Xiaowei Liu, Jiancheng Han, Xiaoyan Gu, Lin Sun, Ying Zhao, Tong Yi, Hongjia Zhang, Yihua He

**Affiliations:** ^1^Beijing Anzhen Hospital, Capital Medical University, Beijing, China; ^2^Beijing Key Laboratory of Maternal-Fetal Medicine and Fetal Heart Disease, Beijing Anzhen Hospital, Capital Medical University, Beijing, China; ^3^Key Laboratory of Medical Engineering for Cardiovascular Disease, Ministry of Education, Beijing, China

**Keywords:** fetal cardiovascular abnormality, genetics, left ventricular noncompaction, noncompaction cardiomyopathy, prenatal diagnosis, whole-exome sequencing

## Abstract

**Objectives:** Noncompaction Cardiomyopathy (NCCM) has been classified as primary genetic cardiomyopathy and has gained increasing clinical awareness; however, little is known about NCCM in the fetal population. We aimed to investigate the clinical characteristics and genetic spectrum of a fetal population with NCCM.

**Methods:** We retrospectively reviewed all fetuses with a prenatal diagnosis of NCCM at a single center between October 2010 and December 2019. These cases were investigated for gestational age at diagnosis, gender, left or biventricular involvement, associated cardiac phenotypes, outcomes, and genetic testing data.

**Results:** We identified 37 fetuses with NCCM out of 49,898 fetuses, indicating that the incidence of NCCM in the fetal population was 0.07%. Of the 37 fetuses, 26 were male, ten were female and one was of unknown gender. NCCM involvement biventricle is the most common (*n* = 16, 43%), followed by confined to the left ventricle (*n* = 14, 38%). Nineteen (51%) had additional congenital heart defects, with right-sided lesions being the most common (*n* = 14, 74%), followed by ventricular septal defects (*n* = 10, 53%). Hydrops fetalis was present in 12 cases (32%), of which four were atypical (pericardial effusion only). Sequencing analysis was performed at autopsy (*n* = 19) or postnatally (*n* = 1) on 20 fetuses. Of the 20 fetuses undergoing copy number variation sequencing and whole-exome sequencing, nine (47%) had positive genetic results, including one with a pathogenic copy number variant and eight with pathogenic/likely pathogenic variants. Non-sarcomere gene mutations accounted for the vast majority (*n* = 7). In contrast, sarcomere gene mutations occurred in only one case (TPM1), and no mutations were identified in the three most common sarcomere genes (MYH7, TTN, and MYBPC3) of pediatric and adult patients. Pathogenic/likely pathogenic variants were significantly more frequent in fetuses with congenital heart defects than those without congenital heart defects.

**Conclusions:** Our data demonstrate that fetal NCCM is a unique entity. Compared with pediatric and adult NCCM, fetal NCCM is more prone to biventricle involvement, more likely to be complicated with congenital heart defects, and has a distinct genetic spectrum.

## Introduction

Noncompaction cardiomyopathy (NCCM), which is also known as left ventricular noncompaction, is an increasingly recognized type of cardiomyopathy characterized by prominent left ventricular trabeculations, deep intertrabecular recesses communicating with the ventricular cavity, and a thin and compacted epicardial layer. While NCCM is considered genetic cardiomyopathy by the American Heart Association (1), the European Society of Cardiology categorizes it as unclassified cardiomyopathy (2).

Genetics plays an essential role in NCCM because a genetic cause can be identified in 17–40% of cases in differently sized studies of pediatric or adult patients, with sarcomere genes being the most common mutant genes (3–12). However, these studies applied various criteria for the interpretation of sequence variants, as well as various sequencing strategies, mostly Sanger or gene panel sequencing containing a limited and varied number of genes. Therefore, the exact genetic spectrum of NCCM remains uncertain. Furthermore, these studies were in cohorts that were primarily adults and children, rarely involving fetuses. Consequently, Little is known about the genetic spectrum of NCCM in fetuses, and whether the insights obtained from pediatric and adult patients can be extended to fetal NCCM is uncertain. In addition, since NCCM has seldom been described prenatally, the incidence and clinical characteristics of NCCM in the fetus are also mostly unknown.

The present retrospective study aimed to identify the genetic spectrum of fetal NCCM using whole-exome sequencing (WES) and copy number variation sequencing (CNV-seq), to assess the incidence of NCCM in the fetal population, and to characterize the largest cohort of fetal NCCM clinically. The objective was also to identify new potential candidate genes for NCCM. These findings provide novel insights into the clinical and genetic characteristics of NCCM in the fetal population, a segment of the human population not well studied. These insights may also help to improve the prenatal and postnatal management of NCCM.

## Methods

### Ethics Statement

This retrospective study was approved by the institutional review board of the Medical Ethics Committee of Beijing AnZhen Hospital. All parents agreed to participate in this study and provided signed informed consent.

### Data Collection

We retrospectively reviewed our experience with fetuses diagnosed with NCCM at Beijing Anzhen Hospital, Capital Medical University, from October 2010 to December 2019. Clinical data were collected regarding gestational age at diagnosis, gender, family history, indications for fetal echocardiography, left or biventricular involvement, associated cardiac and extracardiac abnormalities, and pregnancy outcome. Available postnatal echocardiograms and autopsy studies were reviewed to confirm the diagnosis of noncompaction.

### Echocardiographic Data

Due to the lack of fetal criteria, we used postnatal echo findings of NCCM in this prenatal population. According to the Jenni criteria, the diagnosis of NCCM was based on a consensus of re-evaluated echocardiography by two dedicated participating cardiologists (13). The Jenni criteria were also applied in right ventricular noncompaction. Considering the retrospective nature of this study, we first screened fetuses with a diagnosis of NCCM in the report and then reviewed the echocardiographic images to ensure that the patients met the inclusion criteria. Fetuses without complete, high-quality images were excluded from further analysis.

### Copy Number Variation Sequencing and Data Analyzing

Both CNV-seq and WES were done in the setting of a purely research-based protocol. Next generation sequencing–based CNV analysis is increasingly used in clinical testing (14), and CNV-seq, a viable alternative to arrays for detection of CNV (15), was performed using methods as described previously (16, 17). Briefly, RNA-free genomic DNA was isolated from the umbilical cord using the DNeasy Blood and Tissue Kit (Qiagen GmbH, Hilden, Germany) following the manufacturer's protocol. The quality and concentration of DNA from the samples were assessed using the Qubit 2.0 Fluorometer (Thermo Fisher Scientific, Waltham, Massachusetts, USA). A total amount of 1 μg DNA per sample was used as input material for the DNA library preparations. The sequencing library was generated using Truseq Nano DNA HT Sample Prep Kit (Illumina USA) following the manufacturer's recommendations and index codes were added to each sample. The clustering of the index-coded samples was performed on a cBot Cluster Generation System using Hiseq PE Cluster Kit (Illumina) according to the manufacturer's instructions. After cluster generation, the DNA libraries were sequenced on Illumina Hiseq 4000 or Illumina Novaseq (Illumina, Inc., San Diego, CA, USA) and 150 bp paired-end reads were generated. Then, raw image files were processed by the Bcl To Fastq (Illumina) for base calling and generating the raw data. Reads with adaptors and low quality reads (quality score of <20) were removed. Finally, about 5 million sequencing reads per sample were mapped to the NCBI human reference genome (hg19/GRCh37) using the Burrows-Wheeler Aligner and the allocated to 20-kb sequencing bins with 5-kb sliding to achieve a higher resolution for CNV detection. CNV-seq profiles of each chromosome were represented as log2 of the mean CNV of each sequencing bin along the length of the chromosome.

Detected CNVs were evaluated based on literature reviews and the following public databases: DECIPHER (https://decipher.sanger.ac.uk/), DGV (http://dgv.tcag.ca/), the 1000 Genomes Project (http://www.internationalgenome.org/), OMIM (http://omim.org/), ClinVar (http://www.ncbi.nlm.nih.gov/clinvar), ClinGen (https://www.clinicalgenome.org/) and ISCA CNV (https://www.iscaconsortium.org). According to the American College of Medical Genetics (ACMG) standards and guidelines for interpretation of CNVs, CNVs were classified into five categories: pathogenic, likely pathogenic, uncertain significance, likely benign or benign (14).

### Whole-Exome Sequencing, Variant Annotation, Filtering, and Classification

Genomic DNA was extracted from the umbilical cord and parental blood using a Qiagen DNA Blood Midi/Mini kit (Qiagen GmbH, Hilden, Germany). DNA libraries were prepared using an Agilent liquid capture system (Agilent SureSelect Human All Exon V6; Agilent Technologies, Santa Clara, CA, USA) according to the manufacturer's protocol. The size distribution and concentration of the libraries were determined by Agilent 2100 Bioanalyzer (Agilent Technologies) and quantified using real-time PCR. The DNA library was sequenced on Illumina Hiseq 4000 or Illumina Novaseq (Illumina, Inc., San Diego, CA, USA) for paired-end 150 bp reads according to the manufacturer's protocol. Raw image files were processed using Bcl To Fastq (Illumina Inc.) for base calling and generating raw data. Low-quality sequencing reads were filtered out using a quality score of ≥20. The reads were aligned to the NCBI human reference genome (hg19/GRCh37) using the Burrows-Wheeler Aligner. BAM files were subjected to single nucleotide polymorphism (SNP) analysis, duplication marking, indel realignment and recalibration using GATK and Picard.

After variant detection, ANNOVAR was used for annotation (http://wannovar.wglab.org/). Variant frequencies were determined in dbSNP150 (https://www.ncbi.nlm.nih.gov/SNP/), the 1000 Genomes Project, Exome Variant Server (http://evs.gs.washington.edu/EVS) and gnomAD (http://gnomad-old.broadinstitute.org/) to remove common SNP (minor allele frequency > 0.05%). Then, nonsynonymous, splicing, frameshift and non-frameshift variants, as well as variants located in splice sites within 10 base pairs of an exon were prioritized for study. SIFT (http://sift.jcvi.org), PolyPhen-2 (http://genetics.bwh.harvard.edu/pph2), MutationTaster (http://www.mutationtaster.org) and CADD (http://cadd.gs.washington.edu) were used for predicting the pathogenicity of missense variants, while Human Splicing Finder (http://www.umd.be/HSF.html) and MaxEntScan (http://genes.mit.edu/burgelab/maxent/Xmaxentscan_scoreseq.html) were used to evaluate the effects on splicing. Moreover, databases such as OMIM, ClinVar and Human Gene Mutation Database(http://www.hgmd.org) were used to determine variant harmfulness and pathogenicity where appropriate. Pathogenicity of variants was determined according to current ACMG guidelines that recommend classifying variants into five categories: pathogenic, likely pathogenic, uncertain significance, likely benign or benign (18).

About the TTN gene, according to a recent publication on TTN mutations in cardiomyopathies, we only evaluated the pathogenicity of loss-of-function (LoF) variants (nonsense, frameshift, canonical ±1 or 2 splice sites, initiation codon, single or multiexon deletion) and excluded other variants (19). Variants classified as pathogenic or likely pathogenic were considered positive genetic results. Sanger sequencing was used to validate the presence of positive genetic results.

The novel candidate genes are referred to as genes that have not previously been implicated in NCCM or for which the published data to support the NCCM association may not yet be definitive. For variants in novel candidate genes, we relied on model organism data, tolerance of the gene to sequence variation, co-segregation analysis and *in-silico* prediction where possible.

### Statistical Analysis

Categorical variables are presented as frequencies (percentage) and were compared using the Pearson χ2 test or Fisher's exact test. Statistical analysis was performed using SPSS version 23 (SPSS, Chicago, IL). Two-sided *P*-values < 0.01 were considered significant.

## Results

From October 2010 to December 2019, 40 patients with a diagnosis of NCCM in the report were reviewed, and 37 patients fit our inclusion criteria, including five previously published patients with NONO mutations (20). Three cases were excluded for incomplete imaging or lack of evidence for NCCM. The total number of fetuses undergoing ultrasound examinations in our center was 49,898, indicating an overall incidence of 0.07%.

This cohort was composed of 26 males, ten females and one of unknown gender. The median maternal age was 29 (range 20–40) years, and the fetuses were assessed at a median gestational age of 25 (range 20–33) weeks. The indications for referral for fetal echocardiography were suspected ventricular noncompaction detected by ultrasound screening (*n* = 15), congenital heart defects (CHD; *n* = 12), multisystem malformations (*n* = 1), fetal arrhythmia (*n* = 1), cardiac enlargement (*n* = 1), thickened ventricular wall (*n* = 1) and family history of NCCM (*n* = 5) or CHD (*n* = 1) (Supplementary Table 1).

### Pregnancy Outcome

Thirty-three fetuses (89%) were electively terminated after detailed counseling, with confirmation of NCCM at autopsy in 14 cases. The remaining four (11%) cases were born. Of these four born patients, one was lost to follow-up after birth; one with moderate heart failure was lost to follow-up after 7 months old; one survived without clinical symptoms at 2 years old; one survived but had decreased left ventricular function and a dilated left ventricle, and was admitted to hospital for medical treatment when she was 7 months old.

### Clinical Features

Of the 37 fetal NCCM, 16 (43%) were biventricular involvement, and 14 (38%) were confined to the left ventricle. In the remaining seven cases (19%), NCCM was confined to the right ventricle.

Hydrops fetalis was present in 12 cases (32%), of which four were atypical (pericardial effusion only). NCCM was associated with atrioventricular block II and sinus bradycardia in one case, sinus bradycardia in one case, and atrial bigeminy in one case.

Of the 37 fetuses with NCCM, 19 had additional CHD, the most common being the right-sided lesions (*n* = 14; 74%), followed by ventricular septal defects. This included pulmonary stenosis (*n* = 11), ventricular septal defects (*n* = 10), Ebstein's anomaly (*n* = 3), pulmonary atresia (*n* = 3) and right ventricular hypoplasia (*n* = 3) (Table 1 and Supplementary Table 1).

**Table 1:** General information of the entire cohort.

**Gender**		
	Male/Female ratio	2.6:1
	Male	26 (72%)
	Female	10 (28%)
	Unknown	1
**Maternal Age (years)**		
	20–40, median: 29	
**Gestational Age (weeks)**		
	20–33, median: 25	
**Pregnancy Outcome**		
	Terminated	33 (89%)
	Born	4 (11%)
**Noncompaction Involvement**		
	Biventricle	16 (43%)
	Left ventricle	14 (38%)
	Right ventricle	7 (19%)
**Concomitant Cardiac Defects**		19 (51%)
	Pulmonic stenosis	11
	Ventricular septal defect	10
	Persistent left superior vena cava	5
	Ebstein's anomaly	3
	Pulmonary atresia	3
	Hypoplasia of right ventricle	3
	Tricuspid stenosis	2
	Right aortic arch with mirror image branching	2
	Aortic valve stenosis	2
	Others^*^	
**Rhythm Disturbances**		3 (8%)
**Hydrops Fetalis**		12 (32%)
**Genetic Testing**		20 (54%)

* *Represents congenital heart defect that occurs only once, including: vascular ring, tetralogy of Fallot, atrial septal defect, aberrant right subclavian artery, dysplastic tricuspid valve, ectopic ductus arteriosus, right ventricular diverticulum, right ductus arteriosus, abnormal branching pattern of the aortic arch, interrupted aortic arch type A, coarctation of aorta and anomalous origin of left pulmonary artery from ascending aorta*.

### Genetic Results

Sequencing analysis was performed at autopsy (*n* = 19) or postnatally (*n* = 1) on 20 fetuses. Of the 20 cases undergoing CNV-seq and WES, nine cases (47%) had a positive genetic result, including one with a chromosomal abnormality and eight with pathogenic/likely pathogenic variants. The genotype and phenotype information of the 9 cases are shown in Table 2.

**Table 2:** Genotype and phenotype of patients with positive genetic results.

**ID**	**NCCM Pattern**	**CHD**	**Gene**	**Variant**	**Zygosity**	**Parental origin**	**Pathogenicity**
**PATHOGENIC/LIKELY PATHOGENIC VARIANTS**							
2	BV	EA; PS; VSD	NONO	NM_001145408.1: c.246_249del, p.P83fs7^*^	Hemizygous	Maternal	Pathogenic
3	LV	PA; VSD; PLSVC; RAAWMIB; RDA	NONO	NM_001145408.1: c.246_249del, p.P83fs7^*^	Hemizygous	Maternal	Pathogenic
4	LV	PS; VSD; RVD; PLSVC	NONO	NM_001145408.1: c.246_249del, p.P83fs7^*^	Hemizygous	Maternal	Pathogenic
6	LV	EA; ASD; PS; ABPOTAA	NONO	NM_001145408.1: c.471del, p.Q157fs18^*^	Hemizygous	Maternal	Pathogenic
9	BV	NA	PRKAG2	NM_016203.3: c.G1592A, p.R531Q	Heterozygous	De novo	Pathogenic
10	BV	PS	KCNH2	NM_000238.4: c.A1847G, p.Y616C	Heterozygous	De novo	Pathogenic
17	BV	EA; PS; VSD	NONO	NM_001145408.1: c.471del, p.Q157fs18^*^	Hemizygous	Maternal	Pathogenic
36	LV	NA	TPM1	NM_001018005.2: c.G398A, p.R133Q	Heterozygous	De novo	Likely pathogenic
**ID**	**NCCM**	**CHD**	**Copy Number Variant**		**Size (Mbp)**	**Parental Origin**	**Pathogenicity**
**PATHOGENIC/LIKELY PATHOGENIC COPY NUMBER VARIANTS**							
13	LV	VSD	seq[hg19]del(1)(q42.3q44)		12.952	De novo	Pathogenic
			chr1:g.236262500_249214999del^*^				

*ABPOTAA, abnormal branching pattern of the aortic arch; BV, biventricle; CHD, congenital heart defect; EA, Ebstein's anomaly; LV, left ventricle; NCCM, noncompaction cardiomyopathy; PA, Pulmonary atresia; PLSVC, Persistent left superior vena cava; PS, Pulmonic stenosis; RAAWMIB, Right aortic arch with mirror image branching; RDA, Right ductus arteriosus; RVD, Right ventricular diverticulum; VSD, ventricular septal defect*.

* *, This CNV contains 116 genes, among which fourteen genes FH, MTR, HNRNPU, AKT3, RYR2, EDARADD, ZBTB18, GREM2, COX20, CHRM3, NLRP3, FMN2, ACTN2, and SDCCAG8 are associated with diseases in OMIM database. RYR2 gene is known to be associated with cardiomyopathy (arrhythmogenic right ventricular dysplasia-2)*.

Likely-pathogenic and pathogenic variants were identified in genes reported previously in association with NCCM, including NONO (*n* = 5), KCNH2 (*n* = 1), TPM1 (*n* = 1), and PRKAG2 (*n* = 1). Interestingly, no mutations were identified in TTN, MYH7, and MYBPC3; mutations in these three genes together account for more than 50% of the genetic causes in pediatric and adult patients (3, 4, 21).

In the present cohort, fetal NCCM with CHD were more likely to have a positive result than those without CHD (67 vs. 25%; Fisher exact probability test, *p* = 0.17). However, because of the small sample size, this difference must be confirmed in larger cohorts. When considering the known inheritance patterns of eight cases with pathogenic/likely pathogenic variants, five (62.5%) cases with NONO mutations are X-linked recessive and three (27.5%) cases with mutations in KCNH2, TPM1, and PRKAG2 are autosomal dominant inheritance.

Besides the findings presented above, we also detected variants in novel candidate genes (LHX9, CACNA1A, UBQLN4, CACNA1G, and MYH11) for NCCM in five cases (Supplementary Table 2). All these variants predicted to be deleterious in potential candidate genes met the following conditions: (1) the allele frequency in the gnomAD database < 0.00001, (2) were LoF variants, and (3) occurred in genes that have a probability of LoF intolerance score > 0.9, supporting deleterious effect for predicted LoF pathogenic variants (22). Two of the five mutations were *de novo*, and the other three were inherited from one parent. Further research is required to evaluate any of the suggested candidate genes. Finally, we also identified four variants of uncertain significance in cardiomyopathy-associated genes (TNNT2, PRDM16, LDB3, and SDHA) in three fetuses (Supplementary Table 3). All these variants were paternal inherited, rare and putatively damaging variants.

## Discussion

We present the largest fetal series to date, describing the incidence, clinical characteristics, and genetic spectrum of NCCM. The incidence of NCCM in the fetal population was estimated to be 0.07% from our single-center study. We showed that about 43% of fetuses diagnosed with NCCM were biventricular involvement, which was in contrast to the predominant left ventricular involvement seen in adults and children. In this cohort, 51% of cases had significant congenital heart disease, with right-sided lesions being the most common (78%). Of the 20 fetuses undergoing genetic testing, 50% had a positive genetic result. Fetal diagnosis may be associated with more obvious and potentially more severe diseases, which may have lead resulted in different gene mutations being found. For example, in our study, mutations in sarcomere genes are rare. These findings provide novel insights into the clinical and genetic characteristics of NCCM in the fetal population, a segment of the human population not well studied.

Our study's significant strength is that we performed CNV-seq and WES rather than targeted panel sequencing, which only screened genes associated with cardiomyopathies, such as sarcomere genes. This sequencing strategy led to a sweeping landscape of genetic variation of NCCM because of the ability to assess chromosomal abnormalities and identify novel candidate genes, rather than just sequencing the known NCCM genes. This advantage is well illustrated in cases with LoF variants in the NONO gene (Table 2) (20). At the time of initial analysis, NONO was not considered as a cardiomyopathy related gene in any published literature. Recently, LoF variants in NONO have been reported in several males with NCCM and CHD (23–25), and the results from the mouse model suggested that NONO deficiency was associated with developing heart defects (26). Furthermore, the cardiac phenotype of the five fetuses with NONO mutations is highly consistent (NCCM, pulmonic stenosis/atresia and septal defects). Taken together, we considered NONO a new gene associated with NCCM and reclassified the variants presented in these fetuses as pathogenic during reassessment at a multidisciplinary team meeting.

NCCM is considered rare, and there is no data on its incidence in the fetal population in previous literature. The estimated incidence of fetal NCCM from our single-center study was 0.07%, similar to that in the adult population. Interestingly, there was an increasing trend in the annual incidence of fetal NCCM in our center during the study (data not shown), probably because of increased awareness, improved imaging technology, and increased popularity of prenatal ultrasound screening in mainland China. This phenomenon indicates that the actual prevalence of NCCM may be higher than previously recognized.

**Figure 1:**
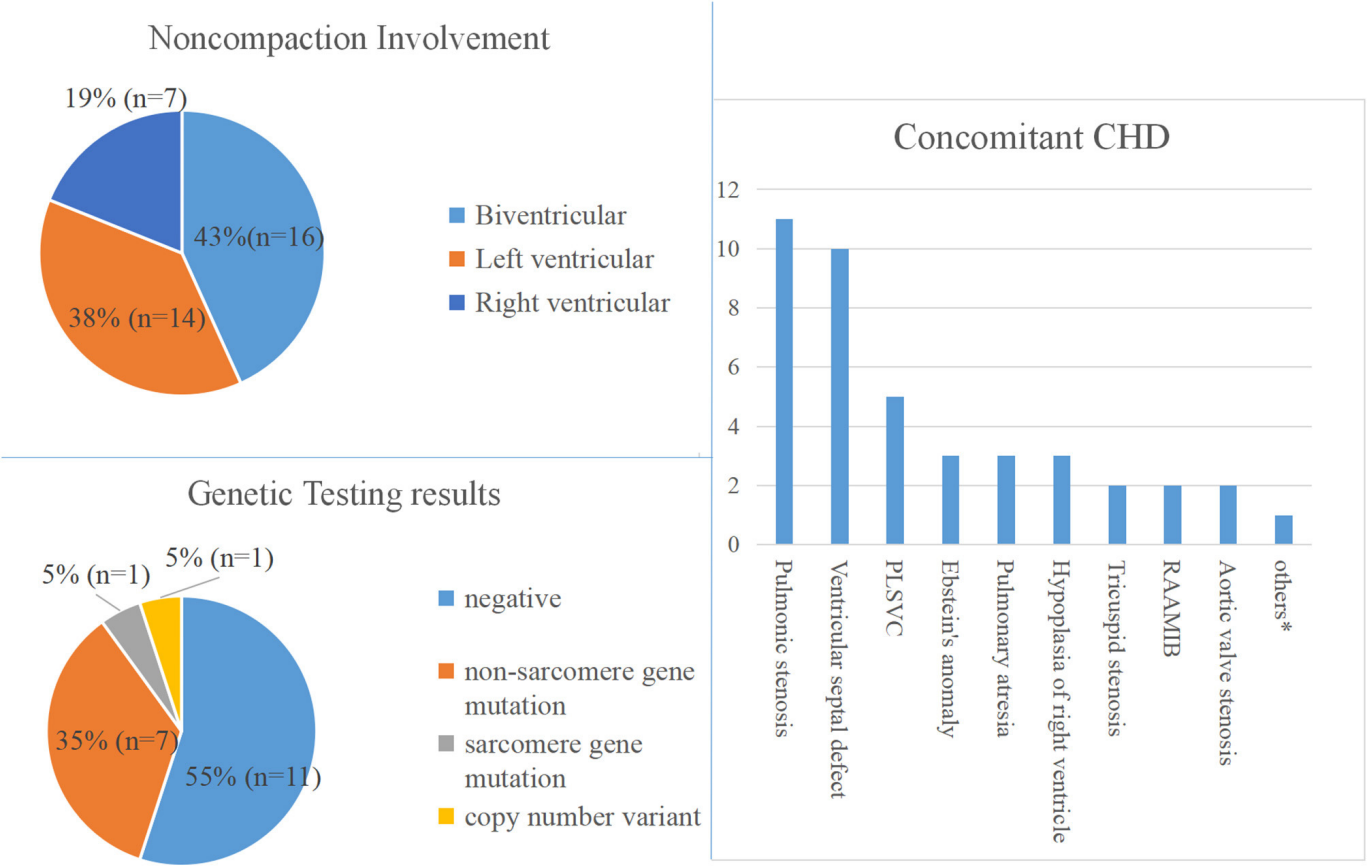
Phenotype and genotype of fetal NVM. PLSVC, Persistent left superior vena cava; RAAMIB, Right aortic arch with mirror image branching. *Represents congenital heart defect that occurs only once.

There appears to be a distinct spectrum of gene variants in fetuses with NCCM. Variants in non-sarcomere genes constituted the majority genetic etiology of NCCM, whereas the prevalence of sarcomere gene variants was unexpectedly low (Table 2 and Figure 1). Furthermore, an X-linked recessive inheritance (NONO mutation) occurs more commonly in this fetal cohort. This genetic spectrum is quite different from previous studies in pediatric and adult patients with NCCM. In adult patients with NCCM, mutations in sarcomere genes, especially TTN, MYH7, and MYBPC3, are most commonly detected and account for the majority of genetic etiology of NCCM (3, 4, 11, 12, 21), among which TTN is the most common (8, 21, 27, 28). In contrast, sarcomere gene mutations are less common in children than in adults, especially in TTN, defects of which are rarely observed in children (4–9), whereas an X-linked or mitochondrial inherited defect or chromosomal anomalies are more common in children than in adults (3). Taken together, these data suggest significant differences in the genetic spectrum of NCCM among fetal, pediatric, and adult patients. These differences should be considered in the clinic and when performing molecular diagnostics. Most importantly, these differences evoke the question of whether the insights obtained from children and adults with NCCM can be extended to fetal patients, especially when determining the gene range for a panel sequencing, simultaneously highlighting that the genetic spectrum of fetal NCCM must be evaluated in larger studies.

Concomitant CHD was more prevalent in our fetal cohort than reported in children and adults. Of the 37 fetuses with NCCM, 51% had significant CHD. Other studies in the literature on fetal NCCM support the high incidence of concomitant CHD (29–32). In contrast, CHD's prevalence in adult NCCM is much lower, with a reported frequency of 5–12% (4, 21, 33). There is some suggestion that children with NCCM have an increased risk for CHD compared with adults, with a frequency of 30–27% (3, 4, 9). Altogether, these data indicate that the prevalence of concomitant CHD is highest among fetuses with NCCM, followed by children, and lowest among adults. Part of the reason for this phenomenon may be that associated additional CHD may confer additional risk of poor prognosis for NCCM, and NCCM fetuses with significant CHD may suffer fetal demise or have died before adulthood (32, 34, 35).

The distribution of CHD subtypes varied among studies. In a recent study of fetal NCCM involving 22 cases with significant CHD, double outlet right ventricle was the most common (64%) (32). Of the 19 cases with CHD in our cohort, right-sided lesions were the most common (74%), particularly pulmonic stenosis (58%) (Table 1). Of the 24 adult NCCM with CHD reported by Stähli et al., left ventricular outflow tract abnormalities were the most frequent (46%) (33). These wide variations in the distribution of CHD subtypes may be partly due to the small number of affected patients and the variable characteristics of the cohorts (e.g., isolated LVNC or not, diagnostic criteria, age at diagnosis).

In contrast to the predominant left ventricular involvement of NCCM seen in adults, one observation emerging from this study is the high incidence of biventricular involvement (43%) in fetal patients. The high incidence of biventricular involvement in fetal NCVM has been described in previous fetal NCCM cases and small series and may be related to the right ventricular dominance of fetal circulation (30, 36–39). The difference between the fetus and the adult in the affected ventricles supports the view that the predominance of circulation may play an important role in noncompaction, and NCCM may reflect an impaired adaptation to special hemodynamics rather than an arrest during myocardial embryogenesis (40).

## Study Limitations

This study has some limitations. The sample size was relatively small. Eighty-nine percentage of fetuses had the pregnancy terminated, so the evolution of the phenotypic findings observed in these fetuses is unknown. Besides, subtle dysmorphic features cannot be determined using fetal ultrasound, and some phenotypes, neurodevelopmental disorders in particular, are impossible to determine in the prenatal setting. Since normally the right ventricle is much more trabeculated than the left, the application of left ventricular noncompaction diagnostic criteria to the right ventricle is not rigorous. However, no recognized diagnostic criteria are available for right ventricular noncompaction. To solve the issue, we chose a compromise method. While applying the left ventricular criteria to the right ventricular, we also listed the right ventricular noncompaction/compaction ratio of fetuses diagnosed with right ventricular or biventricular noncompaction in Supplementary Table 1.

## Conclusion

By clinical phenotyping and next generation sequencing of the largest cohort of fetuses with NCCM, we find that fetal NCCM is a unique entity compared to pediatric and adult NCCM: 1. Concomitant congenital heart defects are more prevalent; 2. Noncompaction involves biventricle is much more common; 3. Mutations in sarcomere genes are rarer. These differences evoke the question of whether the insights obtained from children and adults with NCCM can be extended to fetal patients, especially when determining the gene range for a panel sequencing.

## Data Availability Statement

The datasets presented in this study can be found in online repositories. The names of the repository/repositories and accession number(s) can be found below: https://bigd.big.ac.cn/gsa-human/browse/HRA000495.

## Ethics Statement

The studies involving human participants were reviewed and approved by the institutional review board of the Medical Ethics Committee of Beijing Anzhen Hospital. Written informed consent to participate in this study was provided by the participants' legal guardian/next of kin.

## Author Contributions

HZ, HS, and YH designed the study. XH, XW, XZ, YZhan, XL, JH, XG, LS, YZhao, and TY collected the study materials or samples. HS and TY completed the sequencing experiments. HS, XH, and XW collected and aggregated the data. HS, XH, and XW analyzed and interpreted the data. HS and XH wrote the manuscript. All authors contributed to the article and approved the submitted version.

## Conflict of Interest

The authors declare that the research was conducted in the absence of any commercial or financial relationships that could be construed as a potential conflict of interest.
